# An Unfolding Tragedy of Chagas Disease in North America

**DOI:** 10.1371/journal.pntd.0002300

**Published:** 2013-10-31

**Authors:** Peter J. Hotez, Eric Dumonteil, Miguel Betancourt Cravioto, Maria Elena Bottazzi, Roberto Tapia-Conyer, Sheba Meymandi, Unni Karunakara, Isabela Ribeiro, Rachel M. Cohen, Bernard Pecoul

**Affiliations:** 1 National School of Tropical Medicine at Baylor College of Medicine and the Sabin Vaccine Institute and Texas Children's Hospital Center for Vaccine Development, Houston, Texas, United States of America; 2 James A. Baker III Institute for Public Policy, Rice University, Houston, Texas, United States of America; 3 Autonomous University of Yucatan (UADY), Merida, Mexico; 4 Carlos Slim Health Institute, Mexico D.F., Mexico; 5 Olive View-UCLA Medical Center, Los Angeles, California, United States of America; 6 Medecins Sans Frontieres/Doctors Without Borders (MSF), Geneva, Switzerland; 7 Drugs for Neglected Diseases initiative (DNDi), Geneva, Switzerland and New York, New York, United States of America

In North America, Chagas disease (American trypanosomiasis caused by *Trypanosoma cruzi*) was first reported in Mexico in 1940 [1] and in the United States in Texas in 1955 [2]. However, based on ancient mummified remains discovered in the Rio Grande Valley, human *T. cruzi* infection has been present in North America since prehistoric times [3].

*T. cruzi* is a protozoan hemoflagellate that is most commonly transmitted to humans by blood-feeding triatomine bugs followed by autoinoculation [2]. Chagas disease can also be transmitted to man by non-vectorial mechanisms, namely mother-to-child-transmission [4], blood transfusion, and orally through food-borne transmission. When untreated in the acute stage, the disease becomes chronic and up to 30% or more of infected individuals will progress to Chagasic cardiomyopathy or megavisceral disease associated with debilitating morbidity or death. Today, Chagas disease is a leading cause of heart disease among people living in extreme poverty in the Western Hemisphere, especially in Latin America, where it is a major parasitic killer [2].

**Figure 1:**
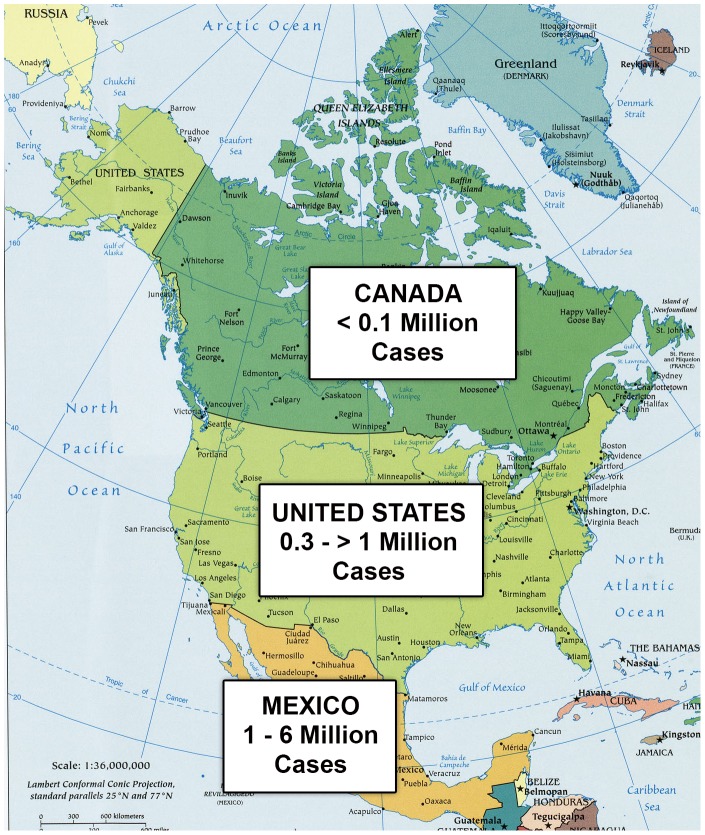
Estimated number of Chagas disease cases in North America.

The established link between poverty and Chagas disease transmission derives largely from poor-quality housing that facilitates triatomine bug invasion, together with lack of access to adequate health care and antenatal care. Additional factors related to poverty also include lack of health education and environmental management leading to vector invasion and colonization [5]. Despite enormous strides made in Chagas vector control, through housing improvements and aggressive insecticidal spraying, and case reduction or even elimination in parts of Latin America [6], important areas of high endemicity persist, including in North America. Confirmatory data are scarce, but, according to some preliminary estimates, Mexico ranks number three, and the United States number seven, in terms of the number of infected individuals with Chagas disease in the Western Hemisphere, where 99% of the cases occur [2]. In Mexico, an earlier national seroprevalence survey reported a rate of 1.6% [7]. However, other reports have provided alternative estimates ranging between 1.0% and 5.9% (i.e., between one to six million cases nationwide) [1], [2]. In the U.S., approximately 300,000 cases are believed to be present [2], although one alternative estimate reports more than 250,000 cases in Texas alone [8], with up to one million or more cases nationwide (Figure 1) [9]. Thus, together with several thousand cases in Canada, there are between 1.5 million (lower estimate) and 7 million (highest estimate) cases of Chagas disease among the 500 million people living in North America. Clearly, there is a need for active surveillance data in order to better refine these prevalence data.

Equally troubling are suggestions that 40,000 pregnant North American women may be infected with *T. cruzi* at any given time, resulting in 2,000 congenital cases through mother-to-child transmission [4]. According to the Pan American Health Organization, congenital transmission may currently account for more than one-quarter of the world's new Chagas disease cases [2], [6].

While North America appears to have a high disease burden resulting from *T. cruzi* infection, in actuality we know remarkably little about the epidemiology of Chagas disease in this region. No systematic effort has been undertaken to ascertain the true national prevalence of Chagas disease in Mexico, even though some isolated communities are now withering because between 30% and 50% of their populations are infected with *T. cruzi* [10]–[12]. In addition, Chagas disease is the most common cause of dilated cardiomyopathy in some areas of southern Mexico [13], and a significant percentage of mothers are infected with *T. cruzi* [14]. According to one estimate the overall economic losses from Chagas disease in Mexico may exceed $3 billion annually [8].

Similarly, in the U.S., no effort to conduct widespread surveillance testing has ever been organized in the states considered at highest risk of *T. cruzi* infection, and, while most of the human cases are presumed to have been introduced through immigration [2], it is known that triatomine vectors infected with *T. cruzi* are present throughout southern Texas and elsewhere in the U.S. [15]. Ironically, we may know more about canine Chagas disease transmission, which is widespread in Texas [16]. While seven autochthonous vector-transmitted human cases in the U.S. had been reported previously [2], the screening of blood donors initiated in 2007 has led to the identification of 16 additional cases [17], but many more may exist, especially given the pervasive ignorance among physicians and other health-care providers on how to recognize and diagnose Chagas disease and how it is transmitted [2], [18]. To underscore the lack of awareness among clinicians, the first congenital case of Chagas disease in the U.S. was reported in 2012, more than 50 years after the first documented case [19].

Alongside the gaps in surveillance is the dearth of health-care facilities offering diagnosis and treatment of Chagas disease in the U.S. Currently, we believe that Los Angeles and Houston have two of the largest clinics for diagnosing and treating the disease. Until recently, treatment of Chagas with benznidazole was thought to be safe and effective only for newborns and infants. The experience of Doctors Without Borders/Médecins Sans Frontières in Latin American countries has shown that treatment with benznidazole is indeed possible—and safe—for children and adults alike, provided regular medical checks are performed [20]. Such activities complement similar, concurrent efforts to evaluate Chagas disease therapies [21], [22]. It is thus imperative to scale up access to benznidazole treatment while simultaneously investing in research for new diagnostics, drugs, and vaccines.

Similar to other endemic countries in Latin America where scaling up appropriate diagnosis and treatment remains a major challenge for Chagas disease patients, the need to address and reduce the burden of Chagas disease in North America is substantial: In Mexico, implementing a national program of surveillance and disease burden assessment is urgently needed, in addition to sustaining vector control efforts, providing accessible programs of case management, and implementing treatment in the most affected and impoverished areas of Mexico (i.e., Chiapas, Guerrero, Jalisco, Oaxaca, Veracruz, and the Yucatan peninsula) [1]. Without such actions, and in the absence of vector control, the Chagas disease burden could double in 25 years [8]. Moreover, in our experience, the waiting list for benznidazole (one of two drugs for the antiparasitic chemotherapy of Chagas disease) can be excessive – therefore a program to accelerate access to this essential medicine needs to be implemented. All pregnant women in Chagas disease-endemic regions of Mexico should be tested for *T. cruzi* infection and offered treatment after delivery and/or after completing breastfeeding. All infants with congenital Chagas disease need to be treated; a new pediatric dosage form of benznidazole developed by the Drugs for Neglected Diseases initiative (DNDi) is now available. In addition, children born during previous pregnancies should also be screened and treated once a mother is identified as infected with *T. cruzi*, as suggested for northern Argentina [23], while overall, new and improved algorithms are needed to address more widespread testing such as testing on women of child-bearing age that migrate from endemic to non-endemic areas.

In the U.S., increased support for federal, state, and local public health agencies is needed to implement a similar program of surveillance, disease burden assessment, and treatment programs in Texas and other southern states, and for some urban communities with large populations of immigrants from Chagas disease-endemic areas. The barriers [24] and cost-effectiveness of immigrant screening, including screening of pregnant women [25], [26], have been noted. Equally urgent is the need to assess the risks of transmission in disease-endemic areas, looking at vectors and reservoir host distributions [1], and to tease out which cases might be transmitted from within the borders of the U.S. There is an urgent need for more, and better, data on human infection in areas where infected vectors and reservoirs have been identified. Regulatory issues surrounding the use of benznidazole in the U.S. must be addressed to provide expanded treatment options. The U.S. is also faced with a health care workforce that now obtains very little, if any, formal training in medical parasitology, so that, at best, physicians are only modestly aware of how to recognize the signs and symptoms of Chagas disease, let alone identify at-risk individuals, obtain clinical testing, and begin appropriate care for patients [2], [18]. Such lack of awareness is especially acute among obstetricians caring for *T. cruzi*-infected expectant mothers [2], [4].

Finally, health research in Mexico needs to be prioritized based on disease burden estimates [27], while research capacity for Chagas disease across North America needs to be expanded to develop or improve current treatments. We desperately require improved drugs, as the two currently available to treat Chagas disease are of limited efficacy in some clinical situations, have undesirable adverse events, and are contraindicated in pregnancy so that we have nothing to offer pregnant women at risk of passing their *T. cruzi* infection onto their unborn fetuses [28]. Neither benznidazole, nor nifurtimox, is approved by the U.S. Food and Drug Administration. DNDi is currently developing a new azole drug candidate that may have a better safety profile, with clinical testing in progress. Concurrently a therapeutic Chagas vaccine is under development by a consortium of Mexican (including the Carlos Slim Health Institute) and Texan scientific institutions [29]. We also need more sensitive diagnostics that can be used at point-of-care, and biomarkers to assess therapeutic response and progression of cardiac disease in the setting of *T. cruzi* infection [2]. Without new tools, disease control will not be possible for Chagas disease, and the U.S. and other governments have a major role to play in ensuring innovation—from basic research to late-stage product development—is funded and carried out.

The poorest people living in the Mexico and the U.S. are silently suffering under a heavy burden of Chagas disease, with pregnant women disproportionately affected. We can save many lives with greater access to the treatments available today, while knowing the fate of tomorrow's patients rests on increasing investments in research to develop new technologies to treat and diagnose Chagas disease, as well as improving scientific cooperation between the U.S., Canada, Mexico, and other key countries. Ultimately, scientific breakthroughs for new technologies to fight Chagas disease will be the cornerstone of efforts to control this ancient scourge in North America and beyond.
